# Hyperpolarized δ-[1- ^13^C]gluconolactone imaging visualizes response to TERT or GABPB1 targeting therapy for glioblastoma

**DOI:** 10.1038/s41598-023-32463-1

**Published:** 2023-03-30

**Authors:** Noriaki Minami, Donghyun Hong, Celine Taglang, Georgios Batsios, Anne Marie Gillespie, Pavithra Viswanath, Nicholas Stevers, Carter J. Barger, Joseph F. Costello, Sabrina M. Ronen

**Affiliations:** 1 Department of Radiology and Biomedical Imaging, University of California San Francisco, 1700 4th Street, San Francisco, CA 94158 USA; 2 Department of Neurological Surgery, University of California, San Francisco, USA

**Keywords:** Diagnostic markers, Brain imaging, Magnetic resonance imaging, Cancer, Cancer imaging, Cancer metabolism, CNS cancer, Tumour biomarkers, Preclinical research, Translational research

## Abstract

*TERT* promoter mutations are a hallmark of glioblastoma (GBM). Accordingly, TERT and GABPB1, a subunit of the upstream mutant *TERT* promoter transcription factor GABP, are being considered as promising therapeutic targets in GBM. We recently reported that the expression of TERT or GABP1 modulates flux via the pentose phosphate pathway (PPP). Here, we investigated whether ^13^C magnetic resonance spectroscopy (MRS) of hyperpolarized (HP) δ- [1-^13^C]gluconolactone can serve to image the reduction in PPP flux following TERT or GABPB1 silencing. We investigated two different human GBM cell lines stably expressing shRNAs targeting TERT or GABPB1, as well as doxycycline-inducible shTERT or shGABPB1cells. MRS studies were performed on live cells and in vivo tumors, and dynamic sets of ^13^C MR spectra were acquired following injection of HP δ-[1-^13^C]gluconolactone. HP 6-phosphogluconolactone (6PG), the product of δ-[1-^13^C]gluconolactone via the PPP, was significantly reduced in TERT or GABPB1-silenced cells or tumors compared to controls in all our models. Furthermore, a positive correlation between TERT expression and 6PG levels was observed. Our data indicate that HP δ-[1-^13^C]gluconolactone, an imaging tool with translational potential, could serve to monitor TERT expression and its silencing with therapies that target either TERT or GABPB1 in mutant *TERT* promoter GBM patients.

## Introduction

Glioblastoma (GBM) remains the most aggressive and treatment-resistant malignant primary intracranial tumor with a very poor prognosis and median survival of 15 months. Upregulation of Telomerase Reverse Transcriptase (TERT) expression resulting from *TERT* promoter mutations is a prominent genetic hallmark of GBM, present in over 80% of GBM patients. As a result, TERT is being investigated as a potential therapeutic target^1–4^. Direct targeting of TERT, the catalytic subunit of telomerase, can affect telomerase activity in normal and germ cells leading to bone marrow suppression and limiting the clinical application of TERT inhibitors^2,5,6^. However, the recent discovery of the TERT upstream transcriptional factor GA-binding protein (GABP) has identified an alternative approach for targeting TERT expression^7,8^. Because GABPB1 is a subunit of GABP, which binds to the mutant *TERT* promoter without interfering with the normal *TERT* promoter, GABPB1 is viewed as a tumor-specific therapeutic approach with potentially limited toxicity^7–10^. Furthermore, recent findings highlight the value of GABP targeting in increasing GBM susceptibility to DNA damage. This identifies the combination of GABP-targeting and classical DNA-damaging agents such as temozolomide as a synergetic therapeutic approach, resulting in the inhibition of tumor growth in vivo^11^. Another related finding discovered the EGFR, AMPK, GABP, and TERT axis in GBM, pointing to additional potential combination therapies for GBM^12^. Collectively, these findings motivated us to develop non-invasive imaging approaches to enable the detection of targeted TERT-silencing and response to therapy.

Previously, we demonstrated the effectiveness of ^1^H-MRS or ^13^C-MRS as useful approaches for assessing TERT or GABPB1-targeted therapies in GBM^9^. Specifically, we showed that ^1^H-MRS-detectable total steady-state lactate, as well as ^13^C-MRS-detectable lactate produced from hyperpolarized (HP) [1-^13^C]pyruvate, are both correlated with TERT expression. However, these metabolic alterations are not specific to TERT. Indeed, changes in ^13^C-MRS-detected HP lactate production have been reported following chemotherapeutic treatments, radiation treatment, and PI3K inhibition^13–17^ and ^1^H-MRS total lactate levels are also affected by the presence of necrotic regions in GBM^18^. We, therefore, wanted to develop additional complementary imaging methods that can further help assess TERT expression and inhibition and enhance the specificity of our imaging.

In our previous study, we found that silencing TERT or GABPB1 in GBM also resulted in a consistent and significant reduction of NADPH and reduced glutathione (GSH). This was associated with a reduction in flux via the pentose phosphate pathway (PPP), which is essential for the production of NADPH and the maintenance of GSH. In a separate study, HP δ-[1-^13^C]gluconolactone metabolism to 6-phosphogluconate (6PG) was reported as a useful probe for monitoring PPP activity^19^. Furthermore, another recent study from our group reported that HP δ-[1-^13^C]gluconolactone crosses that blood–brain barrier and provides a method to distinguish between GBM and normal contralateral brain with higher levels of 6PG detected in tumors^20^. Based on these previous findings, in the present study, we asked whether HP ^13^C MRS imaging of δ-[1-^13^C]gluconolactone metabolism can visualize TERT or GABPB1 modulation. We found that both in cells and orthotopic tumors in rats, silencing TERT expression either directly or upstream at the level of GABPB1 resulted in a significant drop in 6PG production, and 6PG levels were correlated with TERT expression in cells. This identifies another imaging method to monitor response to TERT or GABPB1 targeting therapy in GBM.

## Results

### Expression of pentose phosphate pathway enzymes is dysregulated by TERT or GABPB1 silencing in glioblastoma cells

**Figure 1:**
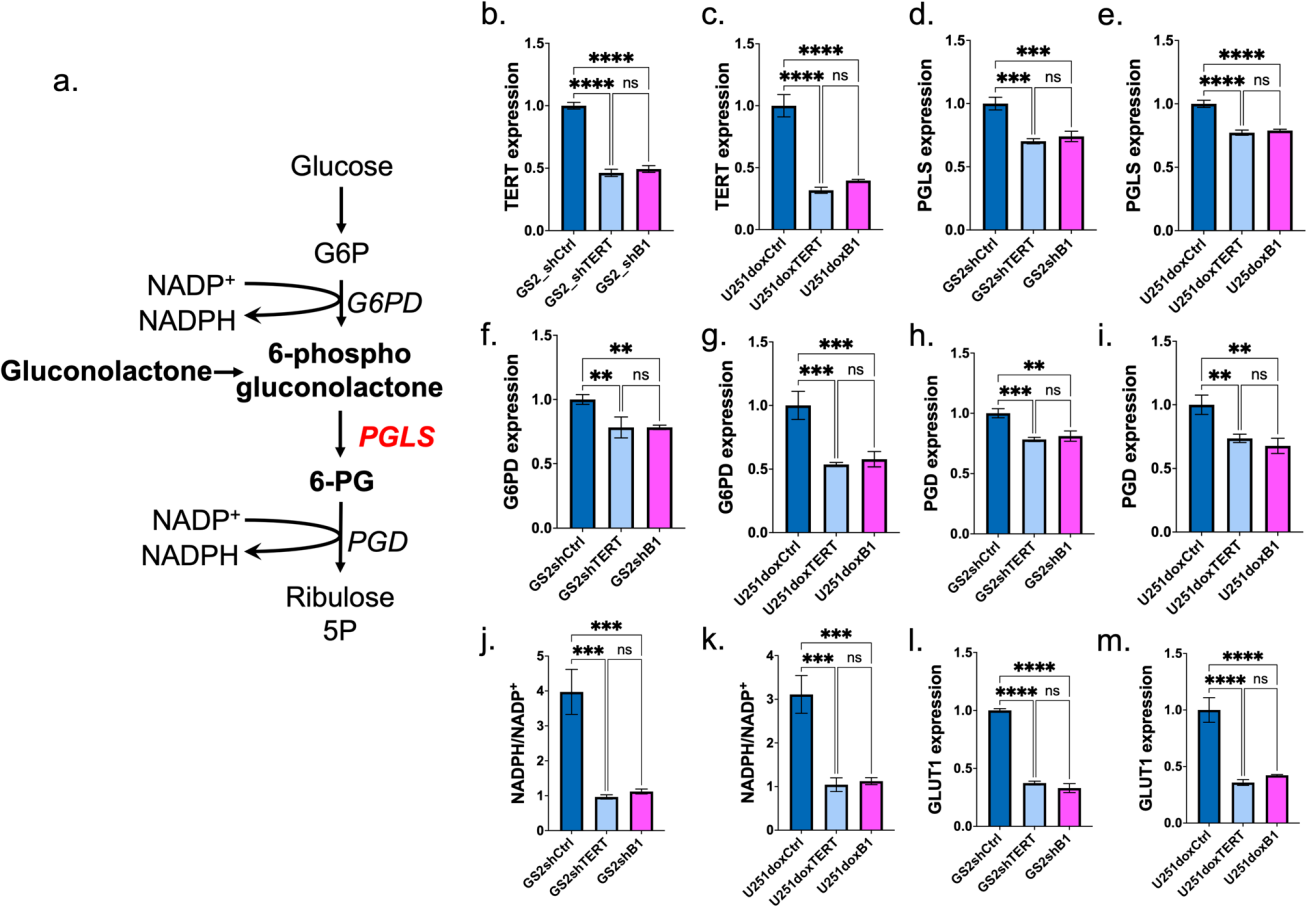
(**a**) Schematic description of pentose phosphate pathway including illustration of the metabolism of gluconolactone and NADPH production. (**b**) TERT expression in GS2shCtrl, GS2shTERT, and GS2shB1. (**c**) TERT expression in U251doxshCtrl, U251doxshTERT, and U251doxshB1. (**d**) PGLS expression in GS2shCtrl, GS2shTERT, and GS2shB1. (**e**) PGLS expression in U251doxshCtrl, U251doxshTERT, and U251doxshB1. (**f**) G6PD expression in GS2shCtrl, GS2shTERT, and GS2shB1. (**g**) G6PD expression in U251doxshCtrl, U251doxshTERT, and U251doxshB1. (**h**) PGD expression in GS2shCtrl, GS2shTERT, and GS2shB1. (**i**) PGD expression in U251doxshCtrl, U251doxshTERT, and U251doxshB1. (**j**) NADPH/NADP + ratio comparing GS2shCtrl, GS2shTERT, and GS2shB1. (**k**) NADPH/NADP + ratio comparing U251doxshCtrl, U251doxshTERT, and U251doxshB1. (**l**) GLUT1 expression in GS2shCtrl, GS2shTERT, and GS2shB1. (**m**) GLUT1 expression in U251doxCtrl, U251doxshTERT, and U251doxshB1.

The PPP is an important source for the production of NADPH (Fig. 1a), which is essential for the maintenance of cellular redox. In this context, our lab recently determined that the activity of the enzyme 6-phosphogluconolactonase (PGLS) is vital in redox maintenance; its knockdown downregulates NADPH and GSH in GBM. PGLS is also central for our HP ^13^C imaging method probing the fate of δ-[1-^13^C]gluconolactone^20^. In the case of U251 cells, we previously showed that the expression of PGLS and other enzymes of the PPP was reduced in TERT or GABPB1-silenced cells (U251shTERT or U251shB1) compared to controls (U251shCtrl)^9^. In the current study, we also assessed the expression of PGLS in our GS2-based model comparing TERT-expressing cells to cells in which TERT expression was silenced by shRNA targeting of either TERT or GABPB1, and in U251cells with doxycycline-inducible TERT or GABPB1 expression (Fig. 1b–e). We also assessed the expression of the other PPP pathway enzymes G6PD and PGD in our models (Fig. 1f–i) comparing TERT or GABPB1 silenced cells to controls. Consistent with our previous results in the U251 cell model^9^, we found that whenever TERT was silenced either directly or via GABPB1-targeting, the expression of all the PPP enzymes was reduced. Most importantly, the enzyme central to our HP ^13^C-MRS approach, PGLS, was reduced whenever TERT expression was reduced. The dysregulation of PPP enzymes in TERT or GABPB1 silenced cells was also associated, as expected, with a significant reduction in NADPH levels that are produced via the PPP, and therefore the NADPH/NADP^+^ ratio in all of our models was significantly reduced when TERT was silenced (Fig. 1j and k).

In our previous work^9^ we had also found that the expression of the glucose transporter GLUT1 is reduced in TERT-silenced cells. Importantly, GLUT1 is also responsible for the transport of gluconolactone into the cell, and can therefore affect HP gluconolactone metabolism to 6PG. We therefore also assessed the expression of GLUT1 in our GS2-based model and in U251 cells with doxycycline-inducible TERT or GABPB1 expression. Consistent with our previous data in the U251 model, we found that TERT silencing led to a drop in GLUT1 expression in every case (Fig. 1l and m).

### HP ^13^C-6PG can detect the effect of stable TERT or GABPB1 silencing

HP δ-[1-^13^C]gluconolactone was described as a method to monitor PGLS activity in the liver^19^ and brain^20^. Based on our finding that PGLS is dysregulated by TERT or GABPB1 silencing, we, therefore, questioned whether the change in PGLS expression, as well as other PPP enzymes and GLUT1, that result from targeting of TERT or GABPB1 could also be monitored using HP δ-[1-^13^C]gluconolactone.

**Figure 2:**
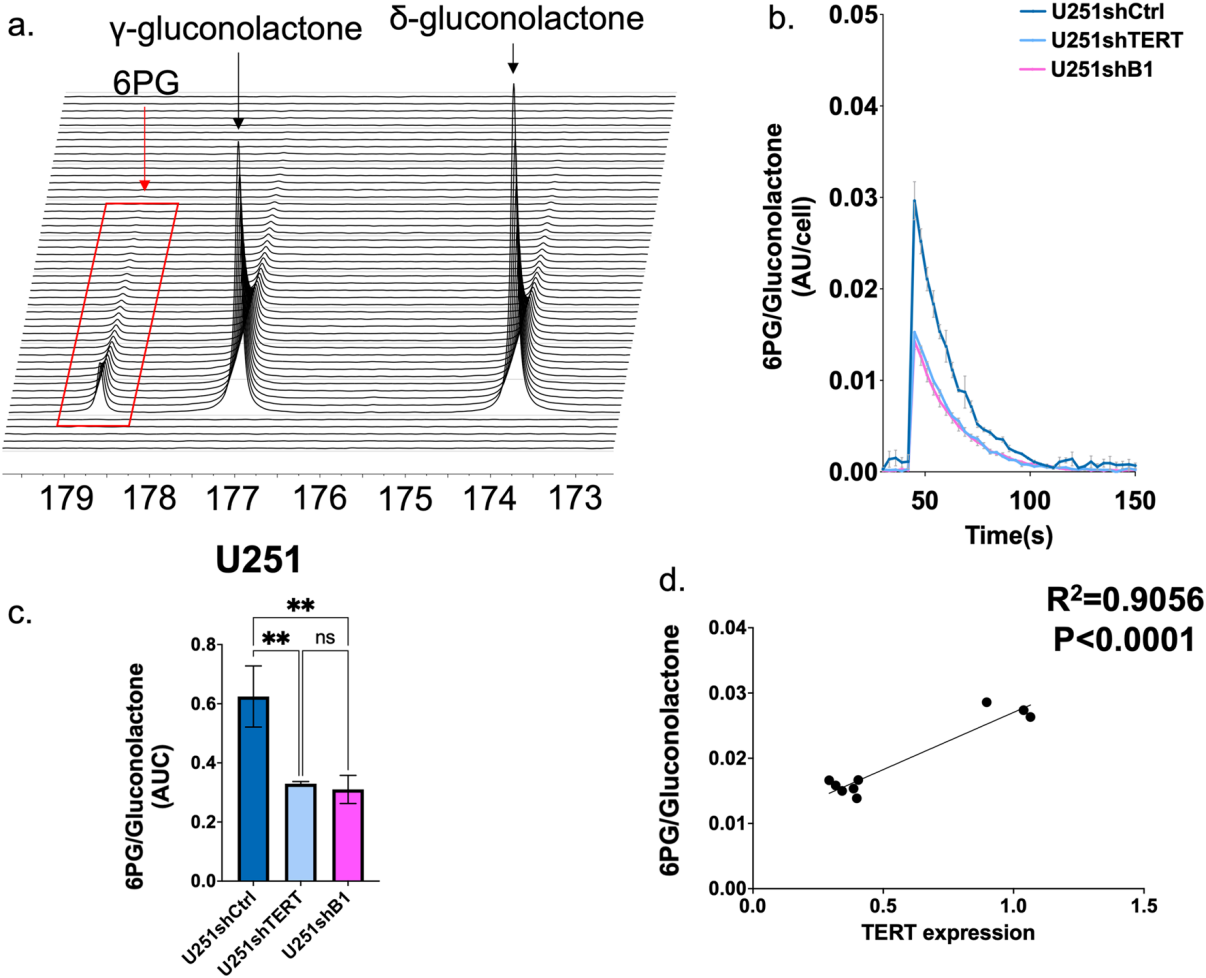
(**a**) Representative spectral array of ^13^C spectra following injection of [1-^13^C] δ-gluconolactone into U251shCtrl cells. (**b**) Temporal evolution of [1-^13^C]6PG comparing U251shCtrl, U251shTERT, and U251shB1 cells. (**c**) The AUC of [1-^13^C]6PG comparing U251shCtrl, U251shTERT, and U251shB1 cells. (**d**) Linear correlation of 6PG levels in U251shCtrl, U251shTERT, and U251shB1 cells as a function of TERT.

First, we wanted to confirm that HP δ-[1-^13^C]gluconolactone and its metabolic product 6PG could be detected in the MRS spectrum of U251shCtrl cells. As shown in Fig. 2a, both metabolites were clearly detected in the ^13^C spectral array of U251shCtrl cells following injection of HP δ-[1-^13^C]gluconolactone. In line with previous studies^20^, γ-[1-^13^C]gluconolactone, which is in rapid equilibrium with δ-[1-^13^C]gluconolactone in the aqueous phase, was also observed. Next, we compared HP δ-[1-^13^C]gluconolactone metabolism between control and TERT-silenced cells. The temporal evolution of [1-^13^C]6PG normalized to total gluconolactone showed that [1-^13^C]6PG levels were suppressed in U251shTERT as well as in U251shB1 cells compared to U251shCtrl (Fig. 2b), and accordingly the quantified AUC of [1-^13^C]6PG normalized to total [1-^13^C]gluconolactone was significantly reduced in U251shTERT and U251shB1 compared to U251shCtrl (Fig. 2c). We also investigated the relationship between TERT expression and relative 6PG production levels. As illustrated in Fig. 2d, although the distribution of points is somewhat limited due to the limited variability in TERT expression in our cells, we observed a correlation between TERT expression and 6PG levels (R^2^ = 0.9056; p < 0.0001).

**Figure 3:**
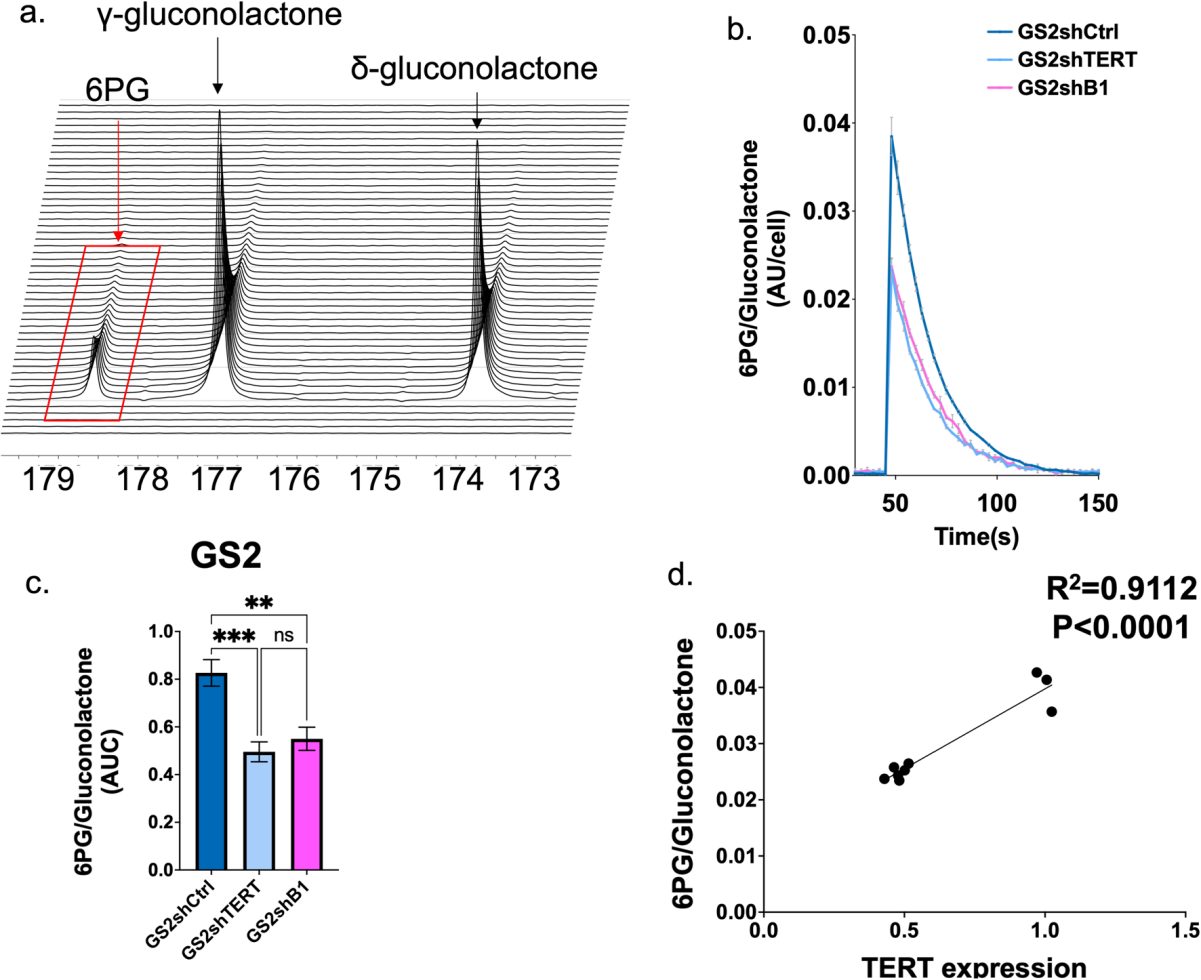
(**a**) Representative spectral array of ^13^C spectra following injection of [1-^13^C]δ-gluconolactone into GS2shCtrl cells. (**b**) Temporal evolution of [1-^13^C]6PG comparing GS2shCtrl, GS2shTERT, and GS2shB1. (**c**) The AUC of [1-^13^C]6PG comparing GS2shCtrl, GS2shTERT, and GS2shB1. (**d**) Linear correlation of 6PG levels in GS2shCtrl, GS2shTERT and GS2shB1 as a function of TERT.

Similar results were also obtained when performing the same experiments and comparing GS2shCtrl, GS2shTERT, and GS2shB1 cells. Again, 6PG levels normalized to total gluconolactone in GS2shCtrl cells were significantly higher than those of GS2shTERT or GS2shB1. A positive correlation between relative 6PG and TERT expression was also observed (Fig. 3a–d).

### HP ^13^C-6PG can detect the effect of rapid TERT or GABPB1 silencing

**Figure 4:**
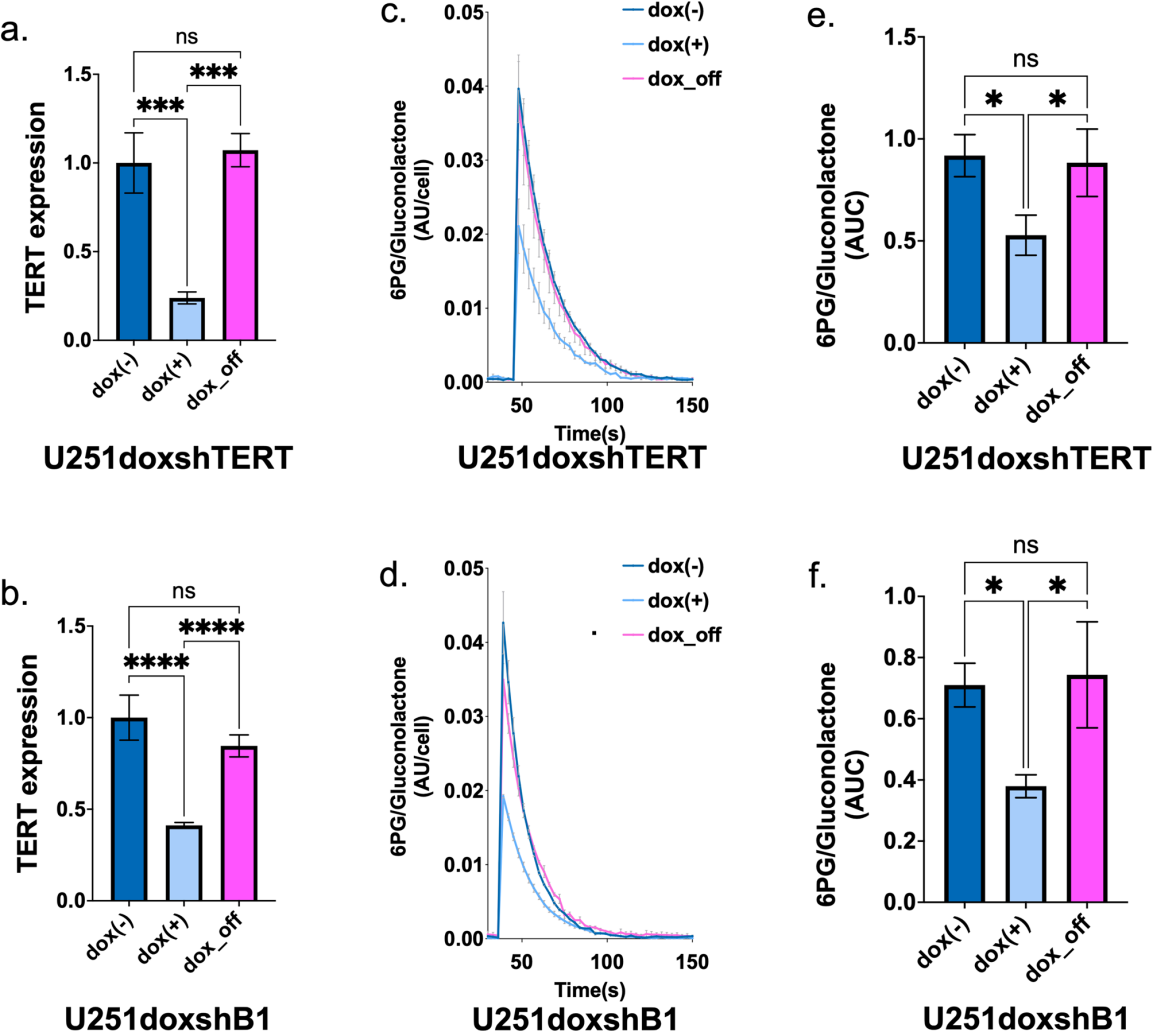
(**a**) TERT expression comparing dox( −), dox( +), and dox_off for U251doxshTERT cells. (**b**) TERT expression comparing dox( −), dox( +), and dox_off for U251doxshB1 cells. (**c**) Temporal evolution of [1-^13^C]6PG comparing U251doxshTERT cells with dox( −), dox( +), and dox off. (**d**) Temporal evolution of [1-^13^C]6PG comparing U251doxshB1 cells with dox( −), dox( +), and dox off. (**e**) The AUC of [1-^13^C]6PG comparing U251doxshTERT cells with dox( −), dox( +), and dox off. (**f**) The AUC of [1-^13^C]6PG comparing U251doxshB1 cells with dox( −), dox( +), and dox off.

Next, we asked whether acute silencing can be rapidly detected by monitoring HP 6PG production. To address this question, we examined two U251 models with doxycycline-inducible shRNA: U251 expressing doxycycline-inducible shTERT (U251doxshTERT), and U251 expressing doxycycline-inducible shGABPB1 (U251doxshB1). First, we confirmed that the expression of TERT was significantly reduced by doxycycline and rescued by the removal of doxycycline in both U251doxshTERT and U251doxshB1 (Fig. 4a and b). Next, we assessed the fate of HP δ-[1-^13^C]gluconolactone by comparing controls (doxycycline-free, dox( −)), to cells exposed to doxycycline for 72 h (dox( +)) and cells 72 h after doxycycline removal (dox_off). As illustrated in Fig. 4c and d, [1-^13^C]6PG production was clearly suppressed by acute silencing of TERT and GABPB1. Importantly, following doxycycline removal, [1-^13^C]6PG levels recovered. The AUC of [1-^13^C]6PG normalized to the maximum total [1-^13^C]gluconolactone confirmed the significant decrease of 6PG levels when TERT or GABPB1 is silenced by doxycycline, and the significant rescued of 6PG levels by doxycycline removal both in U251doxshTERT and U251doxshB1 models (Fig. 4e and f). Collectively, HP ^13^C-6PG imaging can therefore visualize acute TERT or GABPB1 silencing and rescue in our cell models.

### In vivo HP ^13^C-6PG imaging can clearly visualize TERT or GABPB1 silencing in vivo

After confirmation of the utility of HP [1-^13^C]6PG for assessing TERT expression in live cells, our next step was to assess whether we could observe changes in TERT expression after TERT or GABPB1 targeting in preclinical in vivo tumor models.

**Figure 5:**
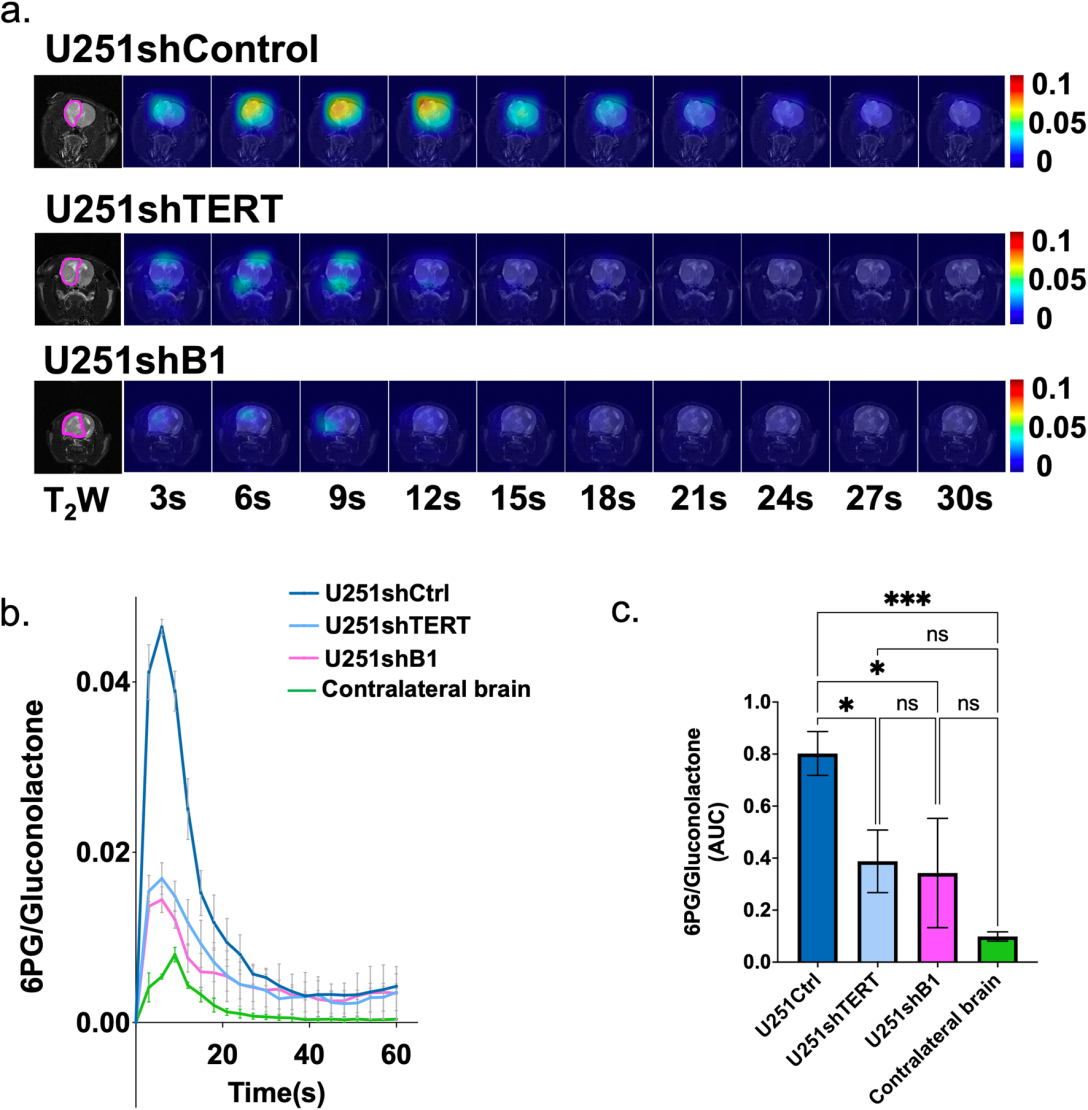
(**a**) Dynamic hyperpolarized ^13^C echo-planar spectroscopic imaging of [1-^13^C]6PG acquired with 3 s temporal resolution. (**b**) Temporal evolution of [1-^13^C]6PG comparing U251shCtrl, U251shTERT, U251shB1 tumors, and contralateral normal brain. (**c**) The AUC of [1-^13^C]6PG comparing U251shCtrl, U251shTERT, U251shB1 tumors, and contralateral normal brain.

First, we probed in vivo tumors formed in rat brains from stably expressing shRNA cell lines, U251shCtrl, U251shTERT, and U251shB. Typically, it took 4–6 weeks to get tumors large enough for the HP MRS data acquisition (> 0.27 mm^3^). Our dynamic HP ^13^C EPSI data set (Fig. 5a), as well as sum spectra over time (Supplementary Fig. S1), clearly indicated that [1-^13^C]6PG production is attenuated in U251shTERT and U251shB1 tumors compared to U251shCtrl, reflecting TERT silencing. Quantification of the dynamic 6PG normalized to the maximum total gluconolactone showed that, as expected, the differences between U251shCtrl and U251shTERT tumors as well as U251shCtrl and U251shB1 tumors are significant (Fig. 5b and c). We also compared our tumor metabolism to metabolism in the normal contralateral brain and found that 6PG levels were significantly higher in tumors when compared to the normal contralateral brain. In fact, the difference between tumor and contralateral brain observed here was greater than observations made in another brain tumor model^20^ possibly reflecting the larger size and greater substrate uptake in our tumor models.

**Figure 6:**
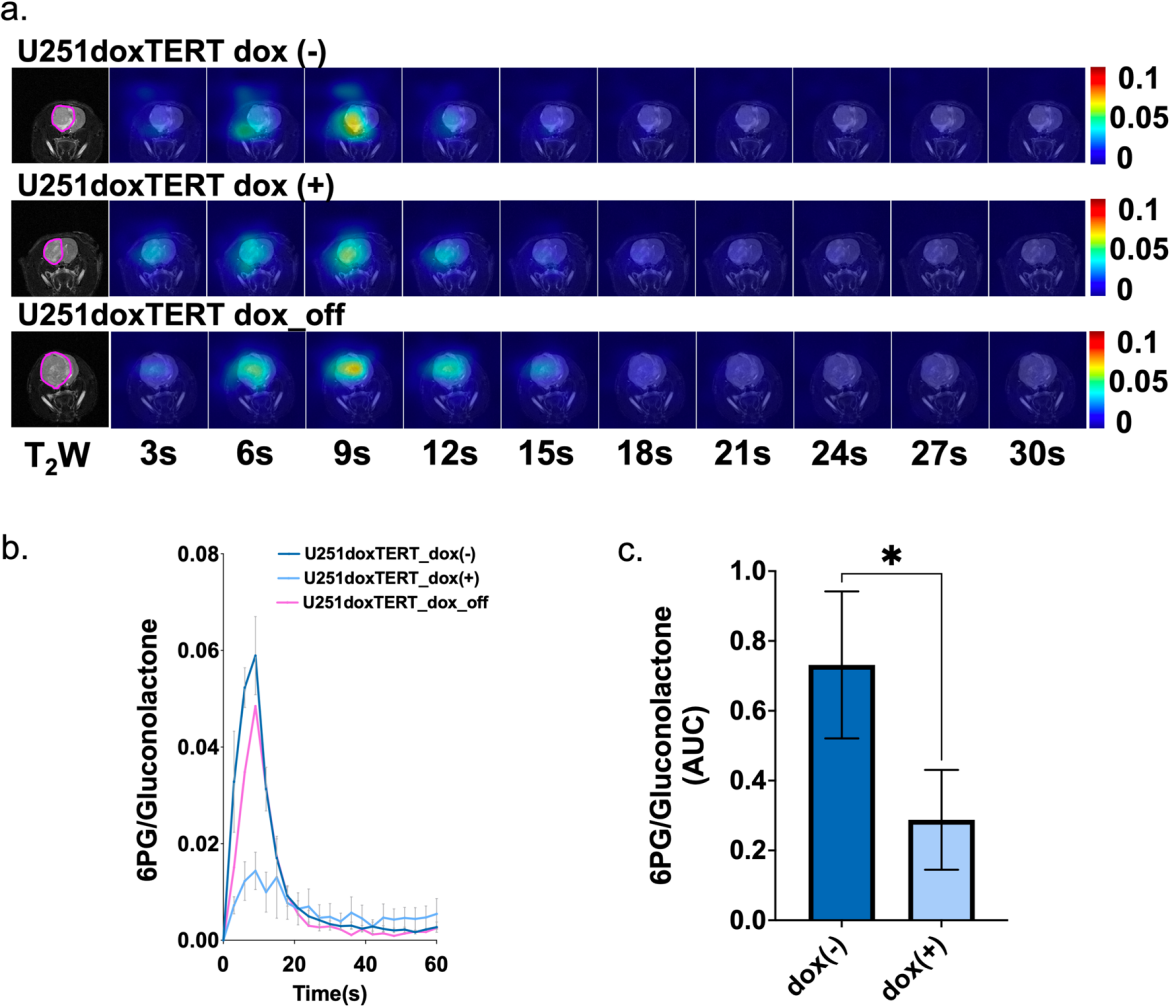
(**a**) Dynamic hyperpolarized ^13^C echo-planar spectroscopic imaging data set of [1-^13^C]6PG acquired with 3 s temporal resolution. (**b**) Temporal evolution of [1-^13^C]6PG comparing U251doxshTERT tumors prior to (dox( −)), following (dox( +)) and after removal (dox_off) of doxycycline. (**c**) The AUC of [1-^13^C]6PG comparing U251doxshTERT tumors prior to (dox( −)) and following (dox( +)) doxycycline.

**Figure 7:**
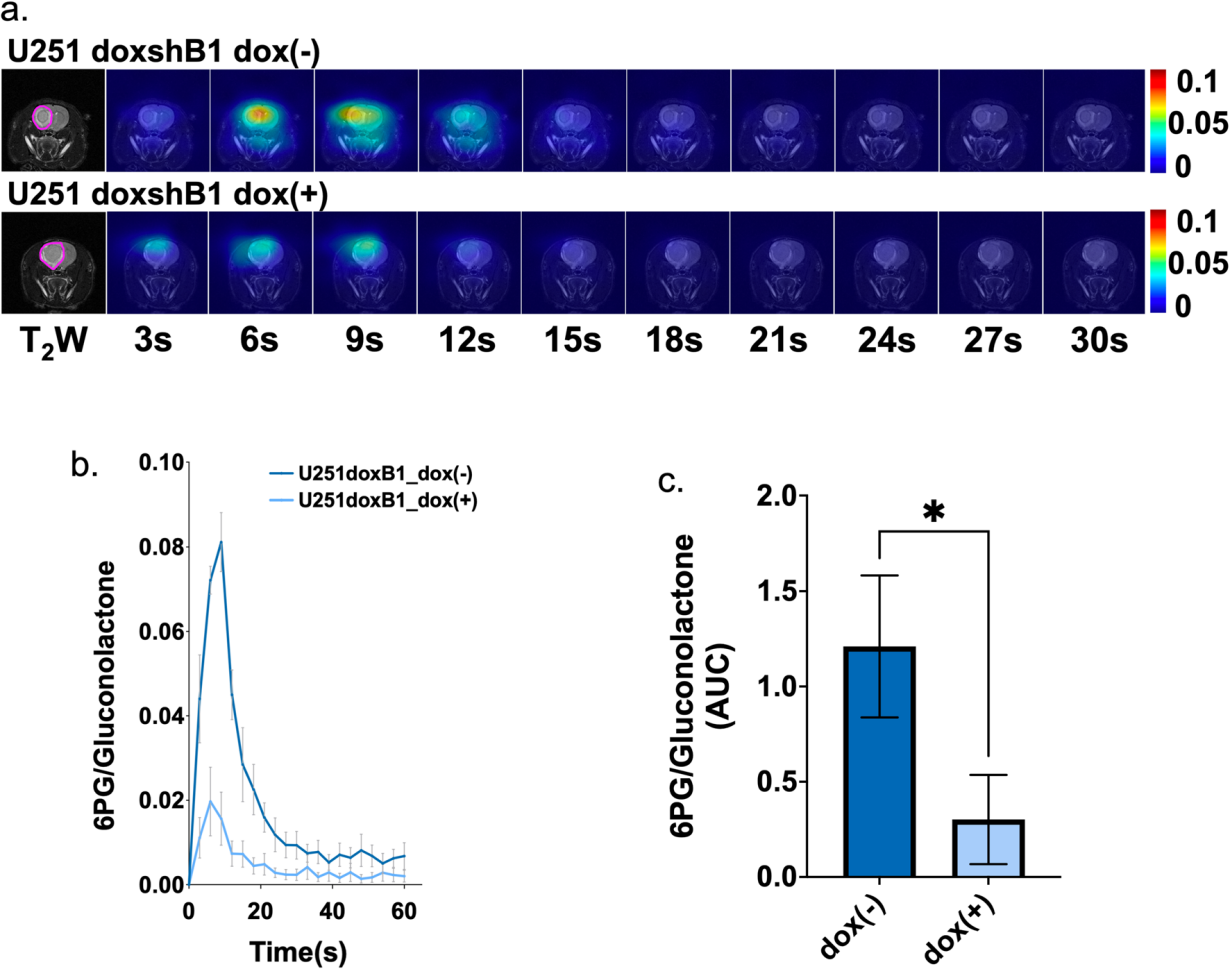
(**a**) Dynamic hyperpolarized ^13^C echo-planar spectroscopic imaging data set of [1-^13^C]6PG acquired with 3 s temporal resolution. (**b**) Temporal evolution of [1-^13^C]6PG comparing U251doxshB1 tumors prior to (dox( −)) and following (dox( +)) doxycycline. (**c**) The AUC of [1-^13^C]6PG comparing U251doxshB1 tumors prior to (dox( −)) and following (dox( +)) doxycycline.

To further confirm our findings and determine whether therapeutic TERT silencing is likely to be rapidly detected in vivo, we also investigated rats implanted with U251 cells expressing doxycycline-inducible shRNAs. We then compared our tumors prior to exposure to doxycycline (dox( −)), following exposure to doxycycline for 7 days (dox( +)), and 14 days after the last day of doxycycline exposure (dox_off, n = 1). The dynamic HP ^13^C EPSI data set of [1-^13^C]6PG normalized to total gluconolactone revealed that 6PG production was reduced by inducible knockdown of TERT via doxycycline in a similar manner to the drop observed when comparing the stable U251shTERT, U251shB1, and U251shCtrl (Figs. 6a and 7a). Quantification of 6PG confirmed a significant decrease in relative 6PG levels in tumors wherein TERT or GABPB1 was modulated by exposure to doxycycline dox( +) (Figs. 6b,c and 7b,c). Removal of doxycycline in a single animal that survived also showed a return of 6PG levels to those observed pre-doxycycline exposure (see Fig. 6a, data not included in Fig. 6c due to lack of statistical significance).

Finally, we also performed a small-scale study on GS2 tumors investigating two GS2shCtrl tumors and one each of GS2shTERT and GS2shB1 tumors in order to validate the results of U251 tumors in a patient-derived model. As expected, quantified 6PG again showed a decrease when TERT or GABPB1 was silenced (Supplementary Fig. S2a–c).

## Discussion

TERT promoter mutations are a hallmark of glioblastoma, enabling TERT expression, telomerase activity and telomere maintenance. Accordingly, TERT and its upstream transcriptional factor GABP are emerging therapeutic targets. Most notably, targeting the GABPB1 subunit is considered a promising approach because GABP is expected to only impact the mutant *TERT* promoter without affecting wild-type *TERT*. As a result, inhibiting GABPB1 would likely have a limited impact on normal proliferating cells and thus potentially limited toxicity^8^. Furthermore, and as mentioned above, recent research has revealed that GABP inhibition increases the susceptibility of GBM to DNA damage, pointing to the potential of combining GABP targeting with traditional DNA damaging agents such as temozolomide for enhanced treatment of GBM^11^. The recent finding of signaling from EGFR via AMPK to GABP and TERT also suggests the possibility of a combination EGFR and GABP targeting as another therapeutic strategy for GBM^12^. Collectively, these recent reports highlight the need for early noninvasive imaging biomarkers of response to TERT silencing in order to achieve better precision medicine for glioblastoma patients.

We previously demonstrated the utility of GSH and lactate in the ^1^H-MRS spectrum, as well as HP lactate production detected by ^13^C-MRS, as biomarkers for TERT-positive glioma and TERT modulation by TERT or GABP silencing. This observation was associated with an overall modulation by TERT of glucose uptake, glycolytic flux, and flux via the PPP. Here we further confirmed that the expression of PPP enzymes and NADPH/NADP^+^ are significantly decreased by TERT or GABPB1 silencing in additional models. In addition, and importantly, we also show that inhibition of the PPP when TERT is silenced can also be noninvasively imaged by probing HP 6PG production using ^13^C-MRS imaging. This finding identifies an additional and complementary approach for noninvasively detecting TERT or GABPB1 silencing, likely enhancing the specificity of our observations.

In this study, we investigated both cells that stably express shRNAs that target TERT or GABPB1, and cells in which shRNA targeting of TERT or GABPB1 is rapidly induced by doxycycline. All these models retained *TERT* promoter mutations that recruit GABP to activate TERT expression. When comparing the contralateral brain to the tumor, and in line with our prior work^20^, we consistently observed higher levels of 6PG in the tumor. Furthermore, when TERT was silenced, either rapidly following doxycycline addition, or in a sustained fashion due to stable shRNA expression, we found that upstream GLUT1 levels and downstream PPP enzyme levels were all reduced. This led to a drop in 6PG production in both cells and in vivo tumors. These observations confirm the association between TERT, enzymes that control gluconolactone uptake and its PPP flux, and HP 6PG production, and highlight the likely value of HP δ-[1-^13^C]gluconolactone imaging as an early indicator of response to TERT-targeted treatment. Indeed, we also observed a significant positive correlation between TERT and HP 6PG production in our cells. This suggests that we could potentially estimate relative TERT expression levels and further points to the value of our imaging approach for early detection of response to TERT or GABPB1-targeting as well as the development of resistance.

Another HP ^13^C probe that could be used to monitor both the glycolytic pathway and the PPP is HP glucose, previously shown as a useful method for monitoring metabolism in lymphoma, lung, and brain tumors^21,22^. However, HP glucose has a relatively short T_1_ relaxation time (9.5 s at 3 T^22,23^) when compared to gluconolactone (32 s at 3 T^20^) potentially limiting the detection of metabolic products. Importantly, gluconolactone and glucose use the same GLUT transporter to enter the cell^19,20,24^ and gluconolactone is being used as a food additive and is therefore unlikely to show significant toxicity^25^. Translation of HP δ-[1-^13^C]gluconolactone to monitor metabolism in patients has not been demonstrated to date, nonetheless this study, combined with prior work, point to the value of monitoring PPP flux in patients.

When considering clinical imaging, positron emission tomography (PET) is commonly used to assess disease in cancer patients. Previous preclinical studies have reported PET or radioactive antisense oligonucleotides to image TERT^26^, however, to date, no clinical translation of these methods has been reported. In contrast, some recent studies have used T_2_-weighted, perfusion, and diffusion MRI images combined with radiomics to detect IDH and *TERT* promoter status^27–30^. Combining our MR metabolic imaging approaches with these MRI-based and advanced machine learning methods could further enhance the value of noninvasive MR-based personalized imaging for GBM patients.

In summary, our study identified ^13^C-MRS-detectable HP 6PG produced from HP gluconolactone as a metabolic biomarker of TERT and its silencing in human GBM with *TERT* promoter mutations, adding another translatable biomarker to the armamentarium of imaging tools that can help improve the monitoring of targeted therapies and personalized treatment of GBM patients, and more broadly any cancer patients for whom TERT might be considered a therapeutic target.

## Methods

### Cell models and cell culture

TERT or GABPB1 silencing for U251 or GS2 was achieved by shRNA transduction as previously described (U251shCtrl, U251shTERT, U251GABPB1, GS2shCtrl, GS2shTERT, and GS2shB1)^9^. Cells were maintained in a 37℃ incubator at 5% CO2. U251 cells were cultured in DMEM/Ham’s F-12 supplemented with 10% fetal bovine serum (FBS), 2 mM glutamine, and 100U/mL penicillin and streptomycin. GS2 cells were cultured in DMEM supplemented with 10% FBS, 2 mM glutamine, and 100U/mL penicillin and streptomycin. Doxycycline-inducible shRNA cell lines (U251doxshtrl, U251doxshTERT, U251doxshB1) were generated as previously described^9^. These cells were maintained in DMEM/F-12 (Gibco, 11330-032) supplemented as above. To silence TERT expression, cells were exposed to 500 ng/mL doxycycline for 72 h prior to HP ^13^C-MRS experiments, and to turn TERT back on cells were cultured without doxycycline for another 72 h. All cell lines were routinely tested for mycoplasma contamination and authenticated by short tandem repeat fingerprinting within 6 months of any experiment.

### RT-qPCR

Total RNA was extracted from cells by using Rneasy Mini Kit (Qiagen, Hilden, Germany), and portions of (1000 ng) RNA were subjected to reverse transcription using High-capacity cDNA reverse transcription kit (Applied Biosystems). Real-time PCR analysis was performed with POWER SYBR Green Master Mix (Applied Biosystems) in a QuantStudio 5 Real-Time PCR System for Human Identification, 96 well. 0.2 mL, desktop (Applied Biosystems). The amplification protocol was as follows: initial denaturation, 95 °C for 30 s, 40 cycles of 95 °C for 5 s, and 60 °C for 30 s. The threshold cycle value was calculated using the crossing point method, and the abundance of each target mRNA was normalized by that of GUSB mRNA. The primer sequences were as follows: TERT_Fwd: TCACGGAGACCACGTTTCAAA, TERT_Rev: TTCAAGTGCTGTCTGATTCCAAT, PGLS_Fwd: CTGCTCACTCTTCCCAGACC, PGLS_Rev: TCCAGTTGCCACAAAGATGA, G6PD_Fwd: CTGTTCCGTGAGGACCAGATCT, G6PD_Rev:TGAAGGTGAGGATAACGCAGGC PGD_Fwd: GGTGCACAACGGGATAGAGT, PGD_Rev: CCATCGGTGTCTTGGAACTT.

### Spectrophotometric assays

NADP^+^ and NADPH levels were measured using a commercially available kit (Biovision, CA). OD450nm was measured using a microplate reader (Tecan, Switzerland).

### HP ^13^C-MRS in live cells

δ-[1-^13^C]gluconolactone was synthesized and polarized as previously described^19,20^. 2 M δ-[1-^13^C]gluconolactone was dissolved in 3:1 water: glycerol and mixed with 15 mM trityl radical OX063. After polarization, samples were dissolved in 6 ml phosphate-buffered saline. 500 ul of HP δ-[1-^13^C]gluconolactone was then injected into ~ 10^8^ live cells in a 10 mm NMR tube to a final concentration of 8 mM. ^13^C-MRS spectra were acquired every 3 s for 300 s on a Varian 500 MHz NMR spectrometer using a 13° pulse and data was analyzed using MNOVA (MestreLab Research, Spain). In line with previous studies, γ-[1-^13^C] gluconolactone, which is in rapid equilibrium with δ-[1-^13^C]gluconolactone in the aqueous phase, was also observed in our spectra and therefore 6PG levels were normalized to maximum total gluconolactone (sum of γ and δ –[1-^13^C]gluconolactone peaks)^19,20^.

### In vivo tumor models

All animal studies were performed in accordance with Institutional Animal Use and Care Regulations and approved by the UCSF Institutional Animal Care and Use Committee (IACUC; approval AN181313). All procedures were performed in accordance with ARRIVE guidelines (https://arriveguidelines.org/). Every effort was made to minimize the number of animals investigated and a total number of 26 animals was used in this study. Animals were investigated until euthanasia was necessary per IACUC guidelines. Investigators were not blinded. Male RH-Foxn1rnu rats weighing 150–200 g and aged 7–8 weeks (Envigo IN) were used for in vivo tumor studies. U251shCtrl, U251shTERT, U251shB1, GS2shCtrl, GS2shTERT, GS2shB1, U251doxshCtrl, U251doxshTERT, and U251doxshB1 cells (5 × 10^5^) in 10 µl Hank’s balanced salt solution were injected into the right putamen using a 26 gauge Hamilton syringe (Hamilton, NV) and a digital compact stereotaxic frame (Harvard Apparatus, MA)^9^. For doxycycline inducible tumors (U251doxshCtrl, U251doxshTERT, and U251doxshB1), to silence TERT, 50 mg/kg doxycycline was injected intraperitoneally daily for 7 days and doxycycline-containing chow was provided prior to the HP studies. To turn TERT back on, animals were returned to doxycycline-free normal conditions for 14 days.

### HP ^13^C-MRS in vivo

All measurements were performed on a 3 T preclinical scanner (Bruker, Germany), equipped with a quadrature ^1^H-^13^C volume coil (Neos-Biotech, Spain). Tumor growth was monitored via T2-weighted images acquired using a multi-slice spin-echo sequence with the following parameters: echo time (TE) = 64 ms, repetition time (TR) = 3484 ms, matrix = 256 × 256, resolution = 0.13 × 0.13 mm2, slice thickness = 1 mm, and the number of averages = 5. Once tumors reached approximately 0.27 cm^3^, we acquired HP ^13^C MRS data assessing HP [1-^13^C]6PG production from gluconolactone as follows. HP ^13^C studies were performed following injection of 2.5 ml of HP δ-[1-^13^C]gluconolactone via a tail-vein catheter over 12 s. Data were acquired using a spectral-spatial echo-planar spectroscopic imaging (EPSI) sequence with a spatial resolution of 5.375 × 5.375 × 8 mm^3^ (TR = 3 s/NR = 20/flip angles = 15.2° on 6PG, 12° on γ-[1-^13^C]gluconolactone, and 3.4° on δ-[1-^13^C]gluconolactone)^23,31^. For the EPSI data, the SNR of each voxel spectrum at every time point was improved using the tensor denoising^23,31^, and spectra were analyzed by determining the area under the curve (AUC) and 6PG normalized to the maximum total gluconolactone as above. All post-processing and quantification procedures were performed using an in-house Matlab (Mathworks, MA) script.

### Statistical analysis

All studies were repeated 3 times unless otherwise stated. All results represent mean ± SD. Either a student’s t-test assuming unequal variance for two-group comparisons or an ordinary one-way ANOVA with multiple comparisons, were used to assess the statistical significance of differences with p < 0.05 considered significant (* = p < 0.05, ** = p < 0.01, *** = p < 0.001, **** = p < 0.0001).

## Supplementary Information

Supplementary Figures.

## Data Availability

Data generated or analyzed during this study are included in this published article or can be obtained from the corresponding author (sabrina.ronen@ucsf.edu) upon request.
