# Supplementary material for: Hyperpolarized δ-[1- 13C]gluconolactone imaging visualizes response to TERT or GABPB1 targeting therapy for glioblastoma
Source: Sci Rep. 2023 Mar 30;13:5190. doi: 10.1038/s41598-023-32463-1 (PMC10063634; doi:10.1038/s41598-023-32463-1)
Supplement: Supplementary file 1 — Supplementary Figures. [file 41598_2023_32463_MOESM1_ESM.docx]

**Supplementary Information**

 Figure. S1

The sum spectra obtained from a. U251shCtrl, b. U251shTERT, c. U251shB1 tumors, and d. contralateral normal brain, respectively.

Figure. S2

1. Dynamic hyperpolarized ^13^C echo-planar spectroscopic imaging of [1-^13^C]6PG acquired with 3 s temporal resolution.
2. Temporal evolution of [1-^13^C]6PG comparing GS2shCtrl, GS2shTERT, and GS2shB1 tumors.
3. The AUC of [1-^13^C]6PG comparing GS2shCtrl, GS2shTERT, and GS2shB1 tumors.
